# Vibration Control of Time-Varying Delay under Complex Excitation

**DOI:** 10.3390/mi12091081

**Published:** 2021-09-07

**Authors:** Kaiwei Wu, Chuanbo Ren, Yuanchang Chen, Sujuan Shao, Jilei Zhou, Chicheng Ma, Lin Li

**Affiliations:** 1 School of Transportation and Vehicle Engineering, Shandong University of Technology, Zibo 255000, China; sdlgwkw@163.com (K.W.); ssjsdut@sdut.edu.cn (S.S.); zhjl521@sdut.edu.cn (J.Z.); machch@sdut.edu.cn (C.M.); lilin20082007@163.com (L.L.); 2 Structural Dynamics and Acoustic Systems Laboratory, University of Massachusetts Lowell, One University Avenue, Lowell, MA 01854, USA; Y.c.chen1990@gmail.com

**Keywords:** time-varying delay, dynamic vibration absorber, stability, hybrid particle swarm optimization algorithm

## Abstract

The existing available research outcomes on vibration attenuation control for time-delay feedback indicate that, for the delay dynamic vibration absorber with fixed time-delay control parameters, under harmonic excitation, a good vibration attenuation control effect occurs on the vibration of the main system. However, the effect is not obvious for complex excitation. Aiming at the above problems, in a short time interval, a harmonic excitation with the same displacement size as the complex excitation was established. Then, by calculating its equivalent amplitude and equivalent frequency, a harmonic equivalent method for complex excitation was proposed in this paper. The time-delay parameters were adjusted according to the equivalent frequency of harmonic equivalent excitation in real time; therefore, a good vibration attenuation control effect was obtained through the delay dynamic vibration absorber in the discrete time interval. In this paper, research on a time-varying delay dynamic vibration absorber was conducted by taking the two-degree-of-freedom vibration system with a delay dynamic vibration absorber as an example. The simulation results show that the proposed control method can reduce the vibration of the main system by about 30% compared with the passive vibration absorber. This can obviously improve the performance of the time-delay dynamic vibration absorber. It provides a new technical idea for the design of vehicle active frame system.

## 1. Introduction

With the rapid development of control theory, active control technology has been widely applied in practical engineering. In vibration control, a certain time is required from signal acquisition, computer analysis, and processing to actuator operating; therefore, there are inevitable time-delay situations when active control is conducted.

In the industrial production process, time delay has become a non-negligible problem with the continuous improvement of requirements of control accuracy. At present, facing this problem, the technical methods of time-delay elimination and time-delay utilization are mainly adopted.

In the early studies, time delays were treated as adverse factors, in which investigators usually identified and compensated for the time delay, such as the methods of Pade approximation processing and sliding mode control [1,2,3,4,5]. In engineering practice, Su and Tang et al. [6] studied the damping control of active suspension systems with input delay, and proposed a variable substitution method to convert the active suspension systems with input delay into the systems without formal delay [7,8,9,10,11]. Wu and Zhou et al. [12] conducted a study on the active vibration damping control of a concrete pump car arm frame based on the time-delay compensation method. It simultaneously caused a small time delay; however, it caused a waste of energy and reduced the control efficiency of the system, as well as led to instability or even bifurcation of the control system. Therefore, the control system designed using the time-delay elimination technique is rough and often leads to wrong conclusions.

With the rapid development and maturity of active control, the research on time-delay control has been carried out comprehensively, and great progress has been achieved in the research of active vibration attenuation control using time delay. Valášek and Olgac et al. [13,14,15,16] first proposed the use of time delay as an active control term for attenuation control, and the use of a delay feedback control based on absorber displacement. It was shown that the absorber can completely eliminate the vibration of the main system under harmonic excitation with the same frequency as the natural frequency of the system. Moreover, the problem of stability analysis of the main system under the action of a delay absorber was solved by the Nyquist and root trajectory methods. Alhazza et al. [17,18,19] studied the nonlinear free vibration of the beam based on acceleration delay feedback control, numerically simulated the nonlinear vibration of the cantilever beam controlled by nonlinear delay feedback under parametric excitation, and experimentally researched the cantilever beam controlled by delay under active multimode vibration. Mirafzal et al. [20] optimized the delay control parameters of the active vibration control of the cantilever beam using the genetic algorithm and conducted related experimental studies. The experiments showed that this method could achieve a better vibration attenuation effect. Kandil et al. [21] used a delay positive position feedback (PPF) controller to reduce the nonlinear vibration of rotating blade dynamic system. Shao et al. [22] studied the effect of a delay feedback control electrically driven resonator on the stability, amplitude, and cycle of the main system in the microelectromechanical field. Saeed et al. [23] studied the nonlinear delay saturation controller to inhibit the vibration of nonlinear beams. Wang and Hu [24,25] theoretically researched the stability and hopf bifurcation behaviour of the delay dynamic system. Chen and Cai et al. [26,27] studied the active control of delay on the rotational vibration and forced vibration of flexible beams, as well as conducted related experimental studies. Xu et al. [28,29] similarly studied the vibration attenuation mechanism of the delay nonlinear dynamic vibration absorber, as well as the role of delay feedback in the self-parametric dynamic vibration absorber and self-parametric vibration system. Later, with the further development of time-delay active control, time-delay active control has been applied in different engineering fields by many researchers. The results showed that the delay dynamic vibration absorber can effectively broaden the frequency band for vibration absorption of the passive vibration absorber. By giving the appropriate delay control parameters, a good attenuation effect was provided for the single-frequency excitation with a similar natural frequency to the dynamic vibration absorber. Meanwhile, the effectiveness of delay attenuation was verified by experiments.

Although the active control suspension system has found great achievements in theoretical research and practical application, some studies have shown that the dynamic vibration absorber with constant delay feedback control can achieve a good vibration reduction effect in the single-frequency excitation range with a similar natural frequency of the system [30,31,32]. However, the effect of time-delay vibration reduction control under complex excitation is not ideal. Meanwhile, in previous studies, the control parameters of the time-delay dynamic vibration absorber were mainly studied by analyzing the stable state of the system to determine the critical time-delay control parameters. Different time-delay parameters were substituted into the vibration system and analyzed. The system frequency response function was used as the optimization objective to determine the time-delay control parameters. However, external excitation was not taken into account in the solution of delay control parameters. Thus, the optimal delay feedback control parameters could not be obtained.

To solve the above problems, a harmonic equivalent algorithm for complex excitation was proposed in this paper. In this paper, the harmonic excitation behaved identically to the complex excitation in the discrete time interval; moreover, the amplitude and frequency of the equivalent harmonic frequency were obtained. By analyzing the critical stability of the system under the action of equivalent harmonic excitation in the discrete time interval, the control time delay of the absorber was determined. In the continuous time interval, the absorber presented the time-varying delay control mode. In this paper, research on a time-varying dynamic vibration absorber was conducted by taking the two-degree-of-freedom vibration system with a delay dynamic vibration absorber as an example.

## 2. Delay Dynamic Vibration Absorber

### 2.1. Delay Dynamic Vibration Absorber Model

**Figure 1:**
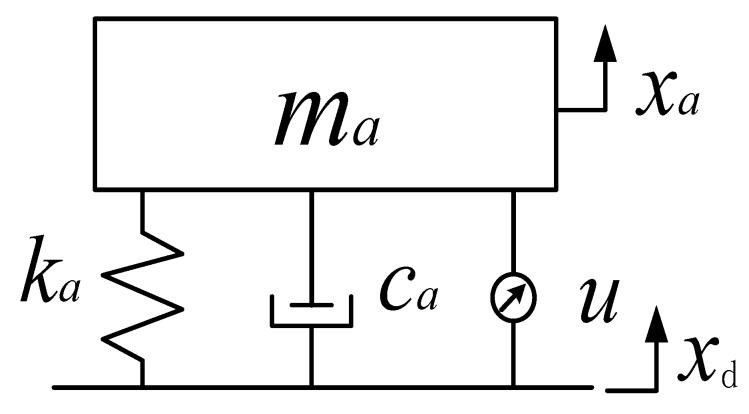
Delay dynamic vibration absorber model.

A dynamic vibration absorber is a kind of equipment that absorbs the vibration energy of the object by using the resonance system to reduce the vibration response of the object. By adding the mass spring resonance system to the vibration object, the reaction force generated by this system when the resonance occurs can attenuate the vibration of the main system. In order to obtain the best attenuation effect of the dynamic vibration absorber, the dynamic vibration absorber absorbs the vibration energy as much as possible under the condition of meeting the stability of the whole system. In engineering practice, the structure and shape of the vibration absorber are complex and variable. For the convenience of research, reasonable simplification of the research object was conducted according to the analyzed points. Figure 1 presents the dynamic vibration absorber model with a time-delay feedback control force after simplification.

The feedback control force $u=gx_{a}\left(t-\tau \right)$ of the delay dynamic vibration absorber takes the displacement of the vibration absorber as feedback, where *g* is the feedback gain, *τ* is the time-delay, *m*_a_, *k*_a_, and *c*_a_ are the mass, stiffness, and damping of the vibration absorber, respectively, and *x*_d_ represents the external excitation.

### 2.2. Critical Stability Parameters of Delay Dynamic Vibration Absorber

When the external excitation is 0, the dynamic equation of the delay dynamic vibration absorber is as follows [33]:

(1) $$m_{a}{\ddot{x}}_{a}+c_{a}{\dot{x}}_{a}+k_{a}x_{a}+gx_{a}\left(t-\tau \right)=0.$$

Carrying out Laplace transform on Equation (1), the characteristic equation becomes

(2) $$m_{a}s^{2}+c_{a}s+k_{a}+ge^{-\tau s}=0.$$

Equation (2) denotes that the transcendental equation has an infinite number of roots, and each characteristic root s=a+ωi has a corresponding time-delay gain *g*. The numerical relationship is expressed as

(3) $$g=\left|m_{a}s^{2}+c_{a}s+k_{a}\right|e^{\tau a},$$

where a is the real part of the characteristic root s.

The result of characteristic Equation (2) satisfies the following angular relationship:

When

(4) $$\begin{array}{ll} ∡(m_{a}s^{2}+c_{a}s+k_{a})=(2k+1)\pi -\tau \omega & g>0\\ ∡(m_{a}s^{2}+c_{a}s+k_{a})=2k\pi -\tau \omega & g<0 \end{array}$$

where k=0,±1,±2,+3,…

From Equation (6), the asymptotic direction of the characteristic root locus of the equation is expressed as follows:

When a→+∞

$$\begin{array}{l} g\to +\infty ,\omega \to (2k+1)\pi /\tau \\ g\to -\infty ,\omega \to 2k\pi /\tau \end{array}$$

When a→−∞

$$\begin{array}{l} g\to 0^{+},\omega \to (2k+1)\pi /\tau \;\\ g\to 0^{-},\omega \to 2k\pi /\tau \end{array}$$

If the root of the characteristic equation of the system is on the left side of the imaginary axis in the complex plane, that is, *a* < 0, the system is stable; if the root of the characteristic equation of the system is on the right side of the imaginary axis in the complex plane, that is, *a* > 0, the system is unstable. For the delay dynamic vibration absorber, when it is in a critically stable state, the vibration reduction effect is the best. Currently, the root of the characteristic equation is a pure virtual root $s=\pm \omega_{c}i$. Substituting pure imaginary roots into Equation (2) yields the critical time-delay parameter. Equation (2) is a transcendental equation; thus, *τ*_c_ is expressed as

(5) $$\tau_{c}=(1/\omega_{c})\{(2l+1)\pi +\tan^{-1}[c_{1}\omega_{c}/(m_{1}\omega_{c}^{2}-k_{1})]\},$$

(6) $$g_{c}=\pm \sqrt{{\left(c_{1}\omega_{c}\right)}^{2}+{\left(k_{1}-m_{1}\omega_{c}^{2}\right)}^{2}}.$$

### 2.3. Research on Damping Efficiency of Delay Dynamic Vibration Absorber

**Figure 2:**
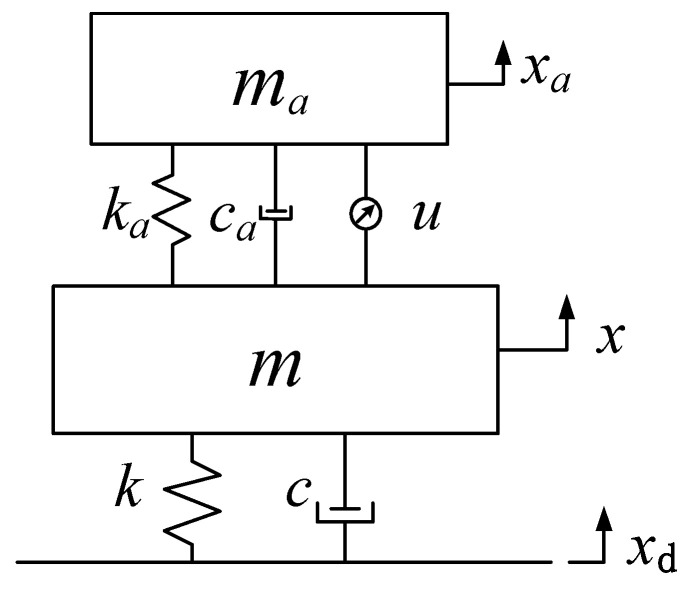
Two-degree-of-freedom vibration system with delay dynamic vibration absorber.

This article contains the two-degree-of-freedom vibration model of the above delay dynamic vibration absorber as an example for research. The model is as shown in Figure 2.

The system dynamics equation of the model is

(7) $$\{\begin{array}{l} m_{a}{\ddot{x}}_{a}+c_{a}\left({\dot{x}}_{a}-\dot{x}\right)+k_{a}\left(x_{a}-x\right)+gx_{a}\left(t-\tau \right)=0\\ m\ddot{x}+c\dot{x}+kx-k_{a}\left(x_{a}-x\right)-c_{a}\left({\dot{x}}_{a}-\dot{x}\right)-gx_{a}\left(t-\tau \right)=c{\dot{x}}_{d}+kx_{d} \end{array},$$

where *m_a_* represents the mass of the vibration absorber, *k_a_* represents the stiffness coefficient of the vibration absorber, *c_a_* represents the damping coefficient of the vibration absorber, *m* represents the mass of the main system, *k* represents the stiffness coefficient of the main system, *c* represents the damping coefficient of the main system, *x_a_* represents the vibration displacement of the vibration absorber, *x* represents the vibration displacement of the main system, and *x_d_* represents the external displacement excitation. The specific parameters are listed in Table 1.

Carrying out Laplace transform on the system dynamics Equation (7), the characteristic equation of the system can be obtained as

(8) $$\left[\begin{array}{cc} A_{11} & A_{12}\\ A_{21} & A_{22} \end{array}\right]\left[\begin{array}{c} X_{1}(s)\\ X_{2}(s) \end{array}\right]=\left[\begin{array}{c} 0\\ F(s) \end{array}\right],$$

where s=ωi, and each coefficient matrix item is

(9) $$\left\{ \begin{array}{l} A_{11}=m_{a}s^{2}+c_{a}s+k_{a}+ge^{-\tau s}\\ A_{12}=-c_{a}s-k_{a}\\ A_{21}=-c_{a}s-k_{a}-ge^{-\tau s}\\ A_{22}=ms^{2}+(c_{a}+c)s+(k_{a}+k) \end{array}\right..$$

From Equation (8), the frequency response functions of the vibration displacement of the vibration absorber and the vibration displacement of the main system are, respectively,

(10) $$\left|H_{1}(\omega )\right|=\left|\frac{X_{a}}{F(s)}\right|=\left|\frac{A_{12}}{A_{21}A_{12}-A_{11}A_{22}}\right|,$$

(11) $$\left|H_{2}(\omega )\right|=\left|\frac{X}{F(s)}\right|=\left|\frac{A_{11}}{A_{11}A_{22}-A_{21}A_{12}}\right|.$$

**Figure 3:**
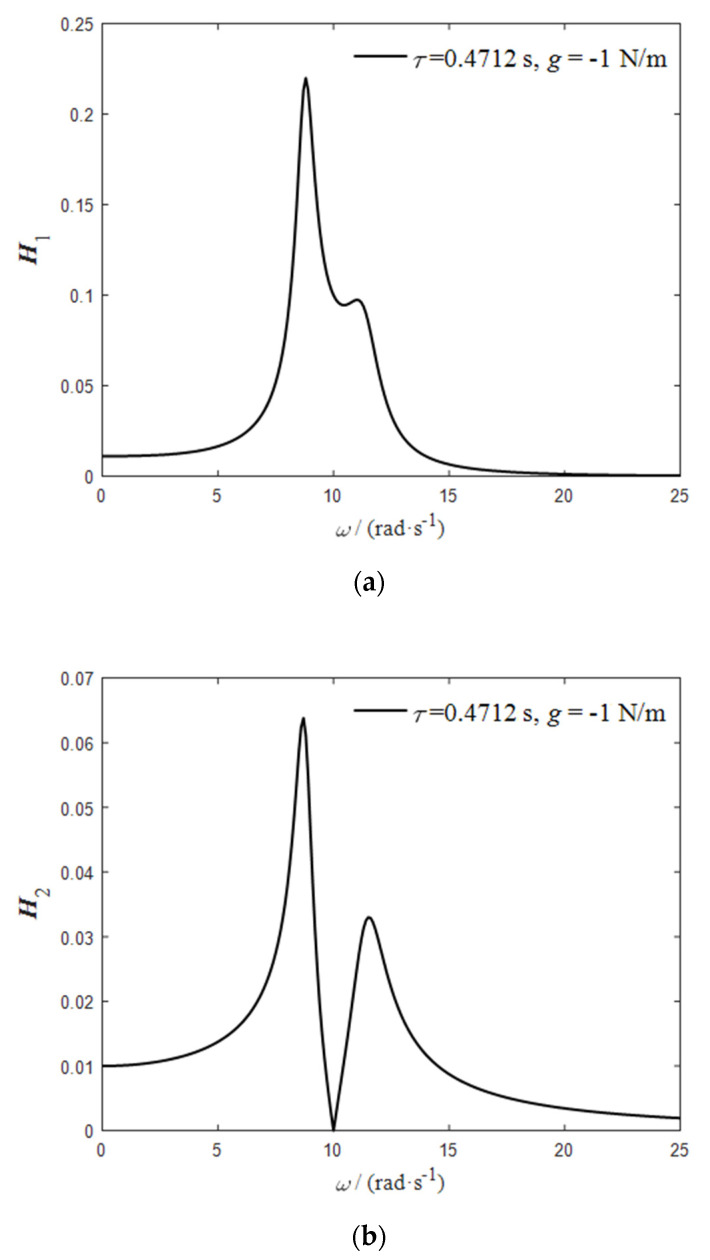
(**a**) Frequency response function curve of delay dynamic vibration absorber; (**b**) frequency response function curve of main system.

Let the external excitation be 0.01 sin (10 t); according to Equations (5) and (6), when the frequency is 10 rad/s, the critical time-delay parameter of the delay dynamic vibration absorber is τ=0.4712,g=−1. Under the action of this pair of time-delay control parameters, the frequency response function curves of the delay dynamic vibration absorber and the main system can be obtained according to Equations (10) and (11), as shown in Figure 3.

**Figure 4:**
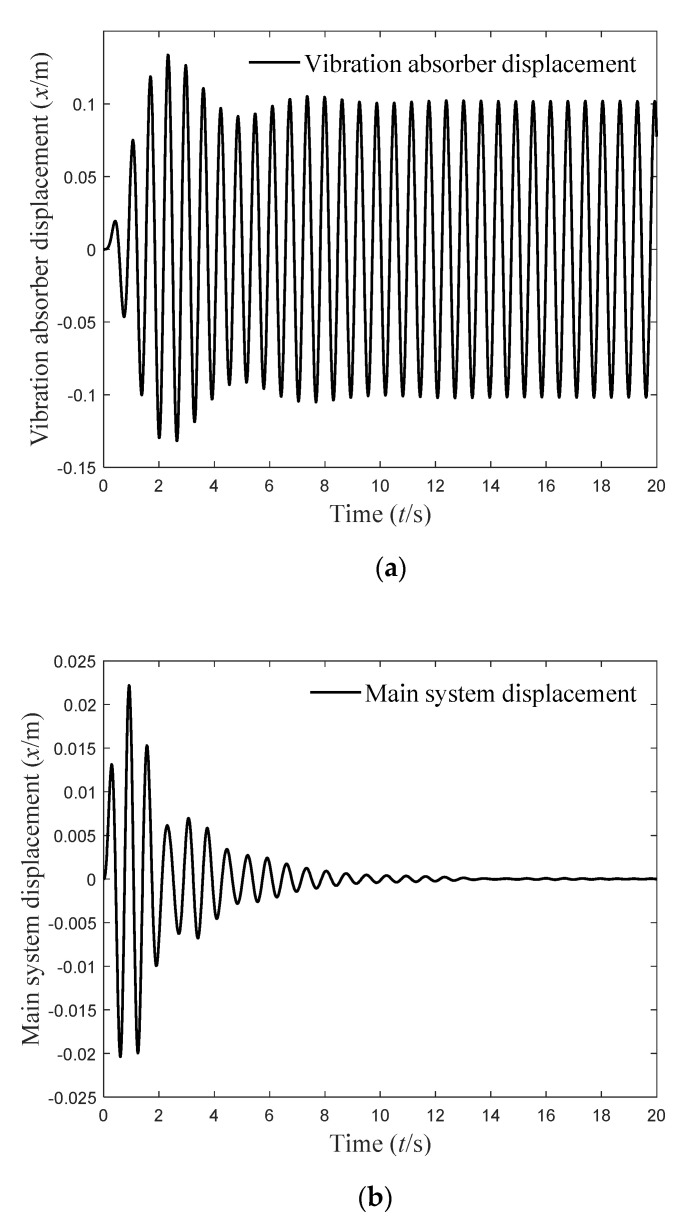
(**a**) Vibration displacement of the vibration absorber; (**b**) vibration displacement of the main system ω=10 rad/s, τ=0.4712 s, g=−1   N/m.

It can be seen from Figure 3 that the frequency domain vibration response of the main system tends to zero at ω=10   rad/s, and the delay dynamic vibration absorber resonates. When ω=10   rad/s, the time-domain vibration response of the delay dynamic vibration absorber and the main system is as shown in Figure 4.

The simulation results show that the time-delay control parameters obtained using Equations (5) and (6) can effectively control the vibration of the main system. In order to verify the generality of the method, the excitation frequency is taken as ω=5    rad/s, ω=8   rad/s, ω=12   rad/s, and ω=15   rad/s, and the obtained time-delay control parameters are, respectively,

$$\left\{ \begin{array}{l} \omega =5\;\;\mathrm{rad}/s,\;\;\tau =0.6150\;\;\;\;s,\;\;g=7.5166\;\;N/m\\ \omega =8\;\;\mathrm{rad}/s,\;\;\tau =0.3654\;\;\;\;s,\;\;g=3.6878\;\;N/m\\ \omega =12\;\;\mathrm{rad}/s,\;\;\tau =0.2840\;\;\;\;s,\;\;g=-4.5607\;\;N/m\\ \omega =15\;\;\mathrm{rad}/s,\;\;\tau =0.2174\;\;\;\;s,\;\;g=-12.5900\;\;N/m \end{array}\right..$$

**Figure 5:**
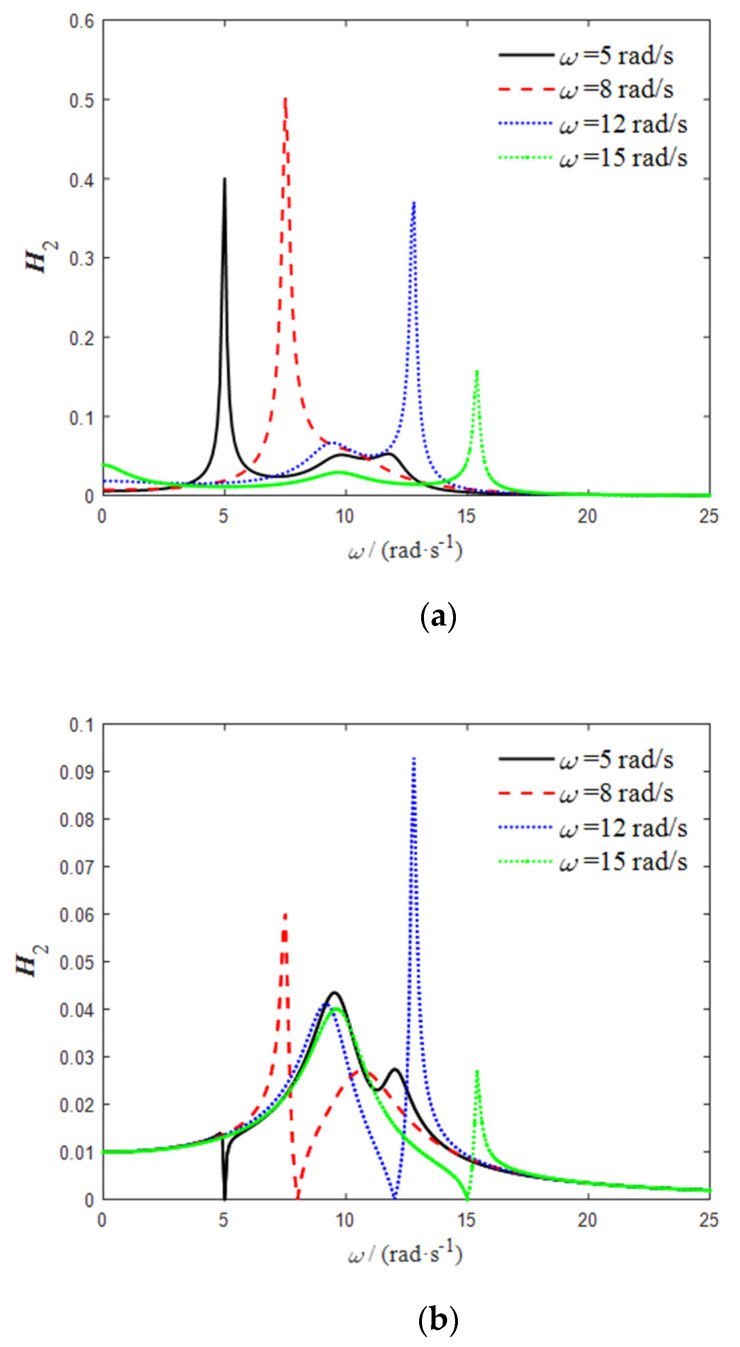
(**a**) Frequency response function curve of delay dynamic vibration absorber; (**b**) frequency response function curve of main system.

Under the action of these four groups of control parameters, the system frequency response function curve is as shown in Figure 5.

As shown in Figure 5, the time-delay control parameters obtained according to Equations (5) and (6) can effectively attenuate the vibration of the main system under different frequencies of external excitation. The absorber is in a resonant state, and the vibration amplitude is large, indicating that the delay control can effectively broaden the absorbing frequency of the dynamic vibration absorber.

## 3. Harmonic Equivalent Method for Complex Excitation

The above outcomes proved the effect of vibration attenuation on the hysteretic dynamic vibration absorber for harmonic excitation. When the complex excitation was known, the complex excitation was replaced equivalently by the changing harmonic excitation. The hysteretic control parameter corresponding to the equivalent frequency of this harmonic excitation was determined in the small-time interval, and then the hysteretic dynamic vibration absorber showed the time-varying delay control in the continuous time interval.

When the complex excitation was known, the harmonic excitation was applied to be equivalent to the complex excitation in the discrete time interval. The continuous time interval was divided into small time intervals by simple harmonic excitation, which was used to approximate the complex excitation in each small segment. When the time division was fine enough, the harmonic equivalent excitation could almost completely approximate the original complex excitation, which proved that the amplitude of the two was the same at each discrete timepoint in the continuous time interval.

Assuming f is a complex displacement excitation, the harmonic equivalent function is l=Asin(ωt), where *A* represents the harmonic equivalent amplitude, and *ω* is the harmonic equivalent frequency, in a small-time interval $\left[t_{k},t_{k+1}\right]$, where

(12) $$\left\{ \begin{array}{l} f\left(t_{k}\right)=l\left(t_{k}\right)\\ f\left(t_{k+1}\right)=l\left(t_{k+1}\right) \end{array}\right.,$$

(13) $$\left\{ \begin{array}{l} A\sin(\omega t_{k})=f\left(t_{k}\right)\\ A\sin(\omega t_{k+1})=f\left(t_{k+1}\right) \end{array}\right.,$$

where $f\left(t_{k}\right)$ and $f\left(t_{k+1}\right)$ are known, and A and ω can then be obtained. In the interval $\left[t_{k},t_{k+1}\right]$, the complex excitation $f\left(t_{k}\right)$ is equivalent to $l=A\sin\left(\omega t_{k}\right)$. The above solution process is carried out for each time interval, and the series of harmonic excitations obtained can be equivalent to the original complex excitations.

**Figure 6:**
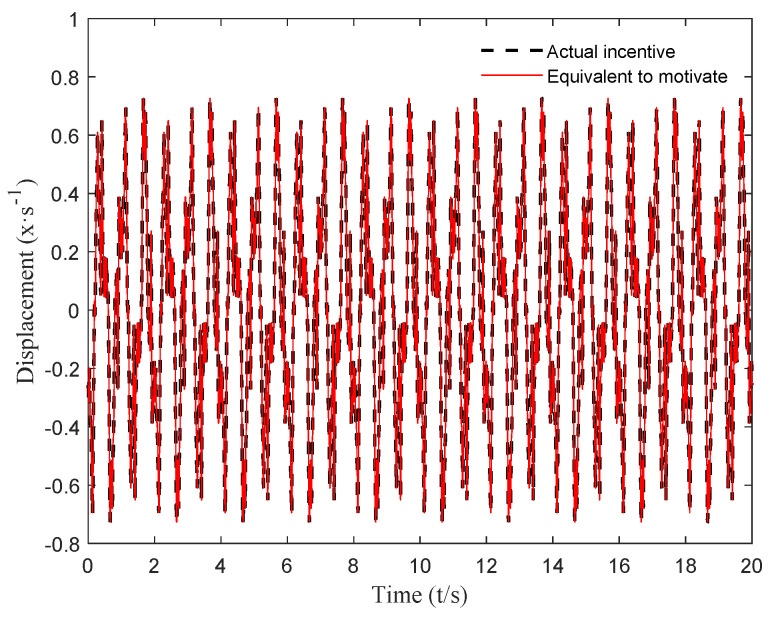
Harmonic equivalent excitation and actual excitation.

In this paper, a complex excitation was constructed using the method in [34]. Suppose the time interval is $\Delta t=t_{k+1}-t_{k}=0.0001\;\;\;s$; then, through the above method, the harmonic equivalent of the constructed complex excitation can be calculated, and the result is shown in Figure 6.

**Figure 7:**
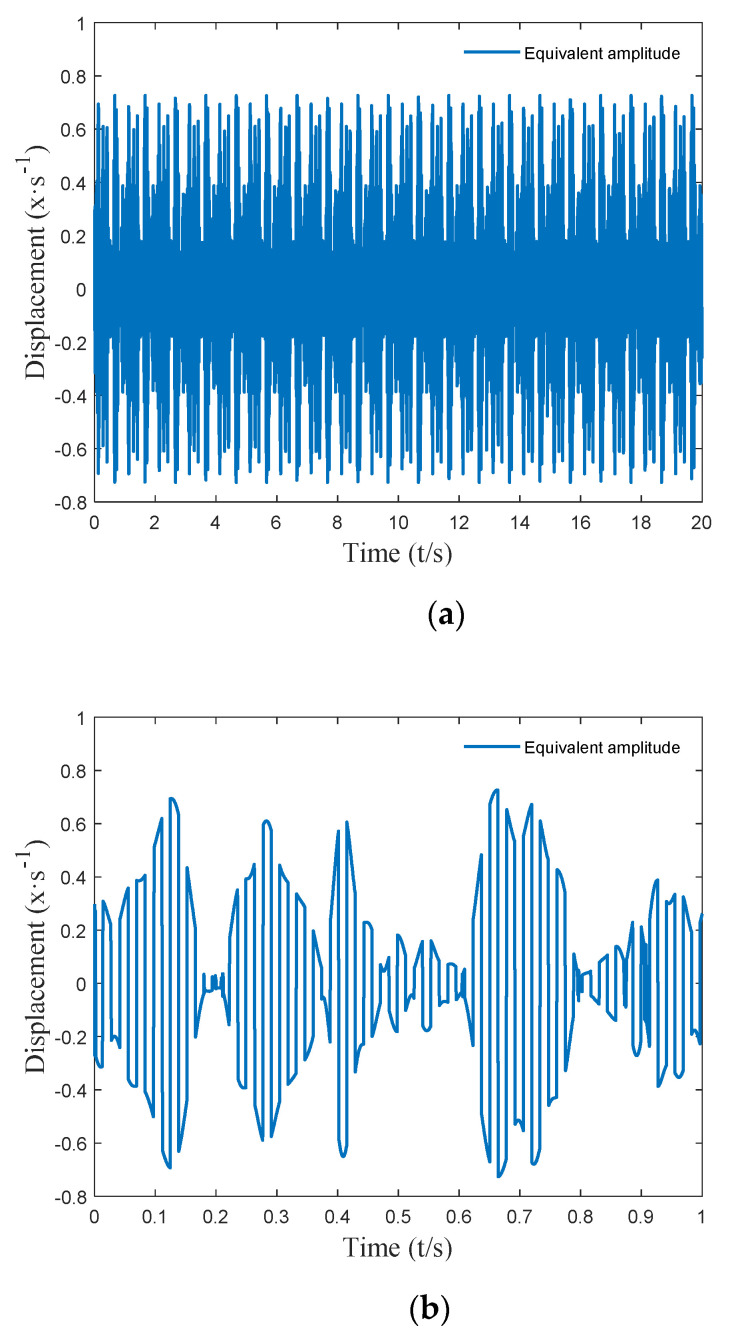
Equivalent amplitude: (**a**) the 20 s amplitude harmonic equivalent excitation effect diagram; (**b**) the 1 s amplitude harmonic equivalent excitation effect diagram.

**Figure 8:**
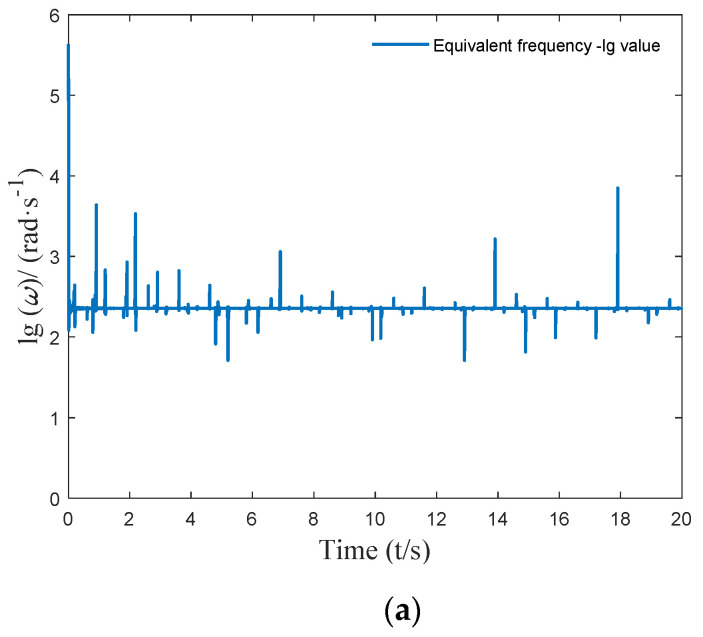

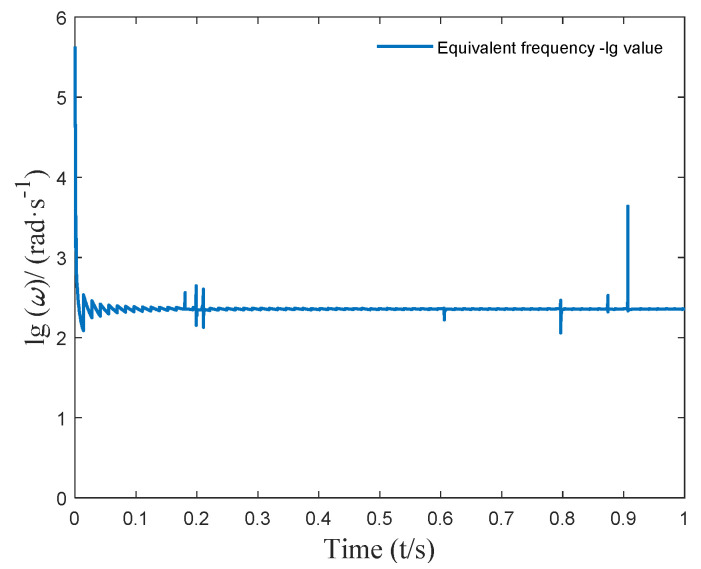
Equivalent frequency: (**a**) the 20 s lg value harmonic equivalent excitation effect diagram; (**b**) the 1 s lg value harmonic equivalent excitation effect diagram.

It can be seen from Figure 6 that the harmonic equivalent excitation obtained using the above method can accurately simulate the actual complex excitation. The equivalent amplitude $A_{e}={\left[A_{1},A_{2}\ldots A_{k}\ldots \right]}^{T}$ and equivalent frequency $\omega_{e}={\left[\omega_{1},\omega_{2}\ldots \omega_{k}\ldots \right]}^{T}$ at each timepoint of the harmonic equivalent excitation are shown in Figure 7 and Figure 8.

## 4. Time-Varying Delay Feedback Control Parameters

### 4.1. Determination of Time-Varying Delay Parameters

**Figure 9:**
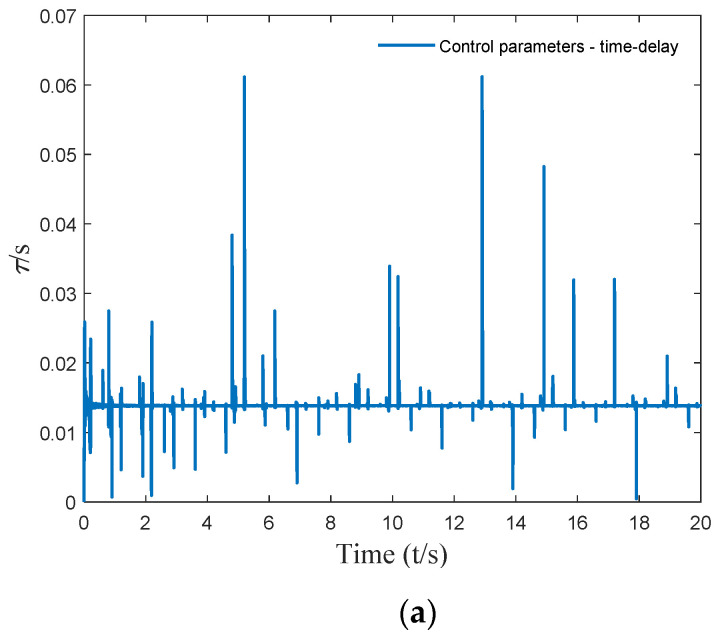

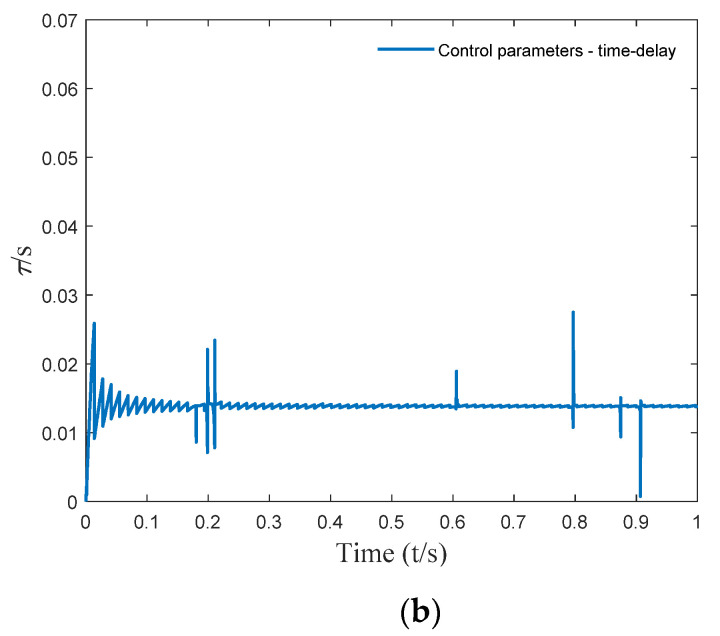
Time-varying delay parameters: (**a**) the 20 s time-delay parameter curve; (**b**) the 0.5 s time-delay parameter curve.

According to the critical time delay solution method, the critical time-delay control parameters corresponding to the equivalent harmonic excitation were calculated, and the results are shown in Figure 9.

In order to minimize the energy consumption during control, this article chose to control the vibration absorber with time-varying delay fixed gain. When the above time-varying delay acts on the delay dynamic vibration absorber, an appropriate fixed time-delay gain *g* is obtained. The time-delay gain *g* is used as an optimization variable, the weighted root mean square of each time-domain response of the main system is selected as the optimization objective function, and the time-delay gain is optimized through natural selection of particle swarm algorithm.

For the two-degree-of-freedom vibration system in Figure 2, the precise integration method [35,36,37,38] was used to solve it, the fine interval was Δt=0.0001   s, and the solution time was t=20   s. Then, each timepoint in 20 s could be used to obtain the time-domain response of the vibration system as follows:

(14) $$\begin{array}{l} \left\{ \begin{array}{l} x={\left[x_{1},x_{2}\ldots x_{m}\ldots x_{n}\right]}^{T}\\ \dot{x}={\left[{\dot{x}}_{1},{\dot{x}}_{2}\ldots {\dot{x}}_{m}\ldots {\dot{x}}_{n}\right]}^{T}\\ \ddot{x}={\left[{\ddot{x}}_{1},{\ddot{x}}_{2}\ldots {\ddot{x}}_{m}\ldots {\ddot{x}}_{n}\right]}^{T} \end{array}\right.\\ m\in \left[1,n\right],\;n=\frac{t}{\Delta t} \end{array}$$

Suppose the optimization objective function is

(15) $$\begin{array}{l} \mathrm{Min}\;J=c_{1}J_{d}+c_{2}J_{v}+c_{3}J_{a}\\ s.t.\;\left\{ \begin{array}{l} g\in \left(g_{\min},g_{\max}\right)\\ \tau =\tau_{s} \end{array}\right. \end{array}$$

where

(16) $$\begin{array}{l} J_{d}=\sqrt{\frac{\sum_{m=1}^{n}{\left(x_{m}\right)}^{2}}{n}}\\ J_{v}=\sqrt{\frac{\sum_{m=1}^{n}{\left({\dot{x}}_{m}\right)}^{2}}{n}}\\ J_{a}=\sqrt{\frac{\sum_{m=1}^{n}{\left({\ddot{x}}_{m}\right)}^{2}}{n}} \end{array}$$

Here, $x_{m}$ is the vibration displacement of the main system at $t_{m}$, ${\dot{x}}_{m}$ is the vibration speed of the main system at $t_{m}$, and ${\ddot{x}}_{m}$ is the vibration acceleration of the main system at $t_{m}$, where $t_{m}=m\times \Delta t$. In Equation (15), *c*_1_ = *c*_2_ = *c*_3_ = 1. The optimal time-delay gain obtained by optimization is *g* = −1.0098 N/m.

### 4.2. Determination of Feedback Gain Based on Hybrid Particle Swarm Optimization Algorithm

Hybrid particle swarm optimization (HPSO) [39] refers to the particle swarm optimization formed by learning from the ideas of some other intelligent optimizations, which naturally obtains the advantages of combining various intelligent algorithms. Combining the characteristics of the optimization objective function in this paper with the characteristics of the high accuracy of the particle swarm optimization based on natural selection, the optimal control gain was quickly obtained by iteration. By combining the natural selection mechanism with the particle swarm algorithm, the particle swarm optimization based on natural selection was obtained. The basic idea was that the entire particle swarm was ranked according to the adaptive value during each iteration, and then the position and speed of the worst half were replaced by those of the best half of the population. Meanwhile, the historical optimal value remembered by each individual was obtained.

Steps of the particle swarm optimization based on natural selection are as follows:

1. The location and velocity of each particle in the random initial population were determined;
2. The fitness of each particle was evaluated, and the current position and adaptation value of each particle in the Pbest of each particle was stored. Then, the position and adaptation value of the individual with the optimal adaptation value was stored in all Pbest in Gbest;
3. The speed and position of each particle were updated;
4. For each particle, the adaptation value and its experienced best position were compared. When the outcome was great, the most current best position was compared;
5. The current values of all Pbest and Gbest were compared, and Gbest was updated;
6. The whole particle population was sorted according to the adaptation value. The position and velocity of the worst half of the particle was replaced with that of the best half in the population; then, the unchanged status of Pbest and Gbest remained;
7. The search was ended when the stop condition was satisfied (usually a preset operation accuracy or iteration times). Then, the result was outputted. Otherwise, the procedure returned to step 3.

In the optimization process, in order to more quickly identify optimal particles, a reasonable range was set in the optimization process according to the established objective function. By writing the algorithm program of a chaotic particle swarm, 80 particles were taken for iterative optimization, the number of particle swarm iterations was 150, the learning factor was taken as 2, the inertia weight was taken as 0.7, and the optimal delay gain g obtained by optimization was −1.0098 N/m.

**Figure 10:**
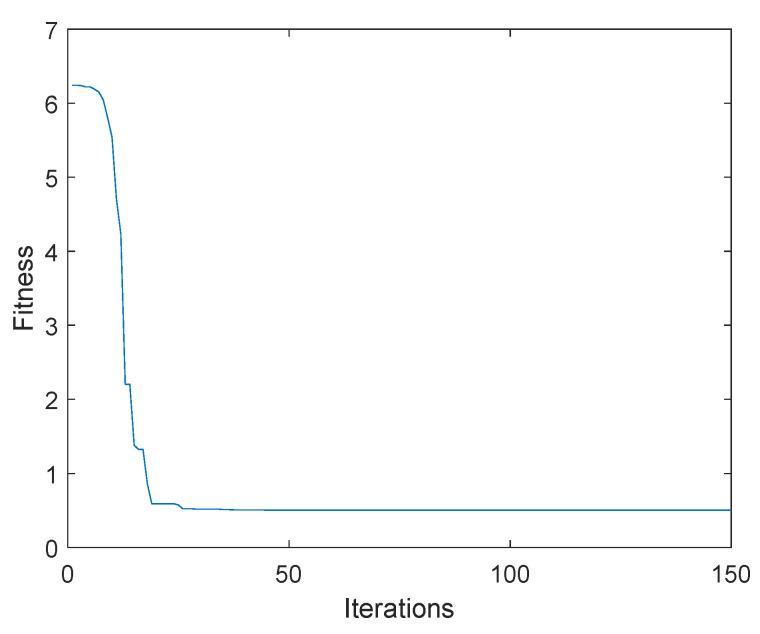
Iterative graph of fitness function.

The change diagram of the iteration optimization of the fitness function was as shown in Figure 10.

## 5. Time-Domain Simulation and Result Analysis

**Figure 11:**
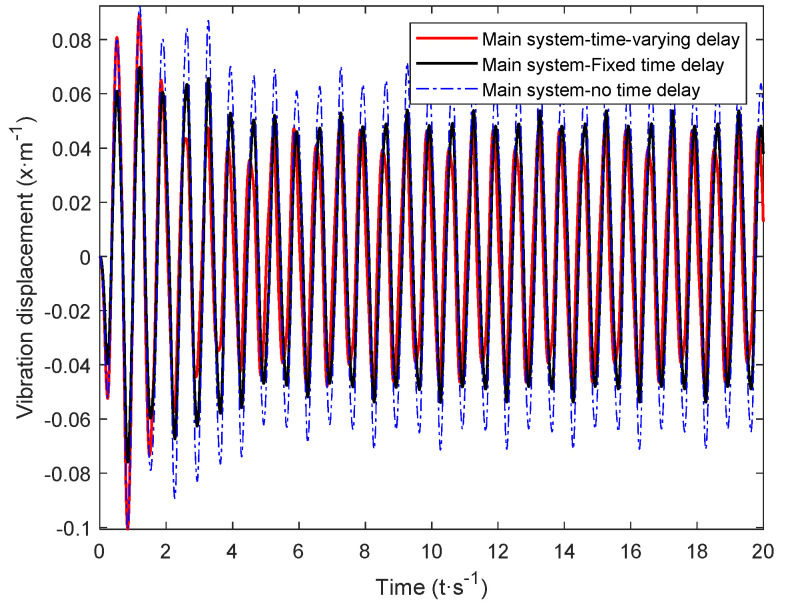
Displacement response of the main system under the action of a time-varying delay absorber.

**Figure 12:**
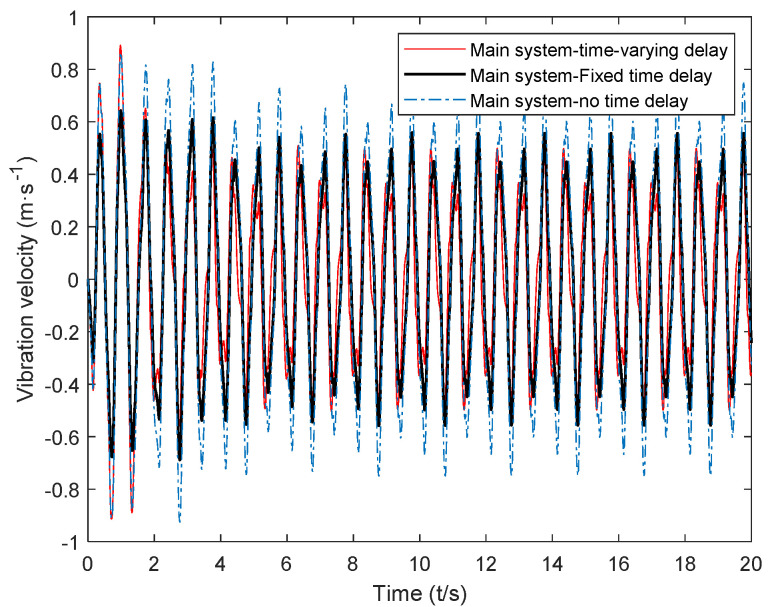
Velocity response of the main system under the action of a time-varying delay absorber.

**Figure 13:**
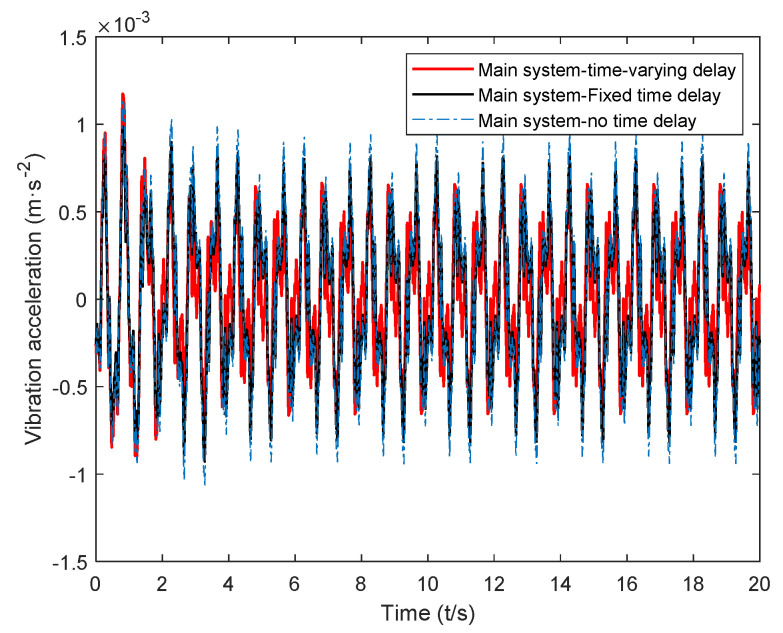
Acceleration response of the main system under the action of a time-varying delay absorber.

The calculated critical time-delay control parameters and the control gain obtained through hybrid particle swarm optimization were introduced into Equation (7). The vibration response of the main system under random excitation was simulated, and the time-varying delay was obtained. The time-domain simulation results of the main system were recorded under the control of the dynamic vibration absorber. Next, the time-delay parameters and the passive vibration absorber were introduced into the system dynamics Equation (7) for calculation, and the time-delay dynamics of the passive vibration absorber and the fixed value parameters could be obtained. The time-domain simulation results of active system vibration under the control of a vibration absorber are shown in Figure 11, Figure 12 and Figure 13.

The time-delay feedback control gain after the time-varying delay and optimization is compared with the vibration response of the main system under the action of a passive absorber and fixed time-delay feedback control in Figure 11, Figure 12 and Figure 13, where it can be seen that, for random excitation, there was a better vibration attenuation control effect in the dynamic vibration absorber under time-varying parameter time delay than the fixed time-delay parameter absorber and passive absorber. During the simulation time of 20 s, the root-mean-square values of each time-domain response of the main system under the action of the two absorbers were calculated (As shown in Table 2). Compared with the passive absorber, under the control of the dynamic vibration absorber with fixed delay control parameters, for the main system, the root-mean-square value of vibration displacement was reduced by 24.53%, that of vibration velocity was reduced by 25.65%, and that of vibration acceleration was reduced by 13.45%. Under the control of the dynamic vibration absorber with time-varying delay parameters, for the main system, that of the vibration displacement was reduced by 34.58%, that of the vibration velocity was reduced by 33.25%, and that of the vibration acceleration was reduced by 22.26%.

From the simulation results, it can be seen that the time-varying delay control parameter method solved using the complex excitation harmonic equivalent method proposed in this paper could significantly improve the vibration attenuation performance of the delay dynamic vibration absorber, and the vibration attenuation effect obtained by the time-varying delay vibration damping control method was consistent with the goal of this paper.

## 6. Conclusions

In this paper, a harmonic equivalent method for complex excitation was proposed. In the transient time interval, the harmonic excitation with the same displacement size was applied to the equivalent complex excitation. The equivalent amplitude and equivalent frequency of equivalent harmonic excitation were calculated. By studying the critical stable state of the absorber, the critical stable time-delay control parameters of the delay absorber corresponding to the equivalent frequency in the transient time were determined. After the time-varying delay was obtained, the fixed time-delay gain was obtained by solving the optimization; thus, the time-delay control parameters of the time-varying dynamic vibration absorber appeared as time-varying time-delay fixed gain parameters in the continuous time interval. The following conclusions were obtained through the simulation experiment:

(1) When the external excitation frequency *ω* was 5, 8, 10, 12, or 15 rad/s, the appropriate time-delay control parameters were selected. The vibration absorber could also achieve a better control effect when the external excitation deviated from the natural frequency of the vibration absorber. Time-delay control could effectively widen the effective damping bandwidth of the vibration absorber.
(2) Time-varying delay feedback control could change the delay control parameters in real time according to the equivalent frequency. In the continuous time interval, the time-delay dynamic vibration absorber behaved as time-varying time-delay fixed gain control. This overcame the problem of active control requiring a large energy input.
(3) The dynamic vibration absorber with time-varying delay obtained using the simple harmonic equivalent method of complex excitation was better than the dynamic vibration absorber with a fixed parameter and passive vibration absorber with time-varying delay. Compared with the passive vibration absorber, the vibration of the main system could be reduced by about 30% under complex excitation. This shows that the time-varying delay dynamic vibration absorber can reduce the vibration of the main system more effectively.

The effectiveness of the proposed time-varying time-delay feedback control strategy is better than that of the passive and fixed time-delay feedback control strategy. It has more engineering application than the feedback control strategy with constant delay. This can provide a new technical idea for the design of vehicle seat active suspension systems.

## Figures and Tables

**Table 1:** Two-degree-of-freedom vibration system parameters.

*m_a_* (kg)	*m* (kg)	*k_a_* (N/m)	*k* (N/m)	*c_a_* (N·s/m)	*c* (N·s/m)
0.1	1	10	100	0.1	2

**Table 2:** Vibration reduction effect of time-varying delay absorber.

RMS	Passive Absorber	Fixed Time-Delay Absorber	Time-Varying Delay Absorber	Vibration Reduction Efficiency of Time-Varying Delay Absorber Relative to Passive Absorber	Fixed Time-Delay Absorber Relative to Passive Absorber
RMS value of main system displacement (m)	0.0470	0.0355	0.0308	34.58%	24.53%
RMS value of main system velocity (m/s)	0.4503	0.3348	0.3006	33.25%	25.65%
RMS value of main system acceleration (m/s^2^)	0.2174	0.1882	0.1690	22.26%	13.45%
